# Tuning Redox Active Polyoxometalates for Efficient Electron‐Coupled Proton‐Buffer‐Mediated Water Splitting

**DOI:** 10.1002/chem.201903142

**Published:** 2019-08-08

**Authors:** Jie Lei, Jun‐Jie Yang, Ting Liu, Ru‐Ming Yuan, Ding‐Rong Deng, Ming‐Sen Zheng, Jia‐Jia Chen, Leroy Cronin, Quan‐Feng Dong

**Affiliations:** ^1^ State Key Laboratory for Physical Chemistry of Solid Surfaces Department of Chemistry College of Chemistry and Chemical Engineering, iChem (Collaborative Innovation Center of Chemistry for Energy Materials) Xiamen University Xiamen Fujian 361005 P. R. China; ^2^ College of Mechanical and Energy Engineering Jimei University Xiamen Fujian 361005 P. R. China; ^3^ School of Chemistry University of Glasgow Glasgow UK

**Keywords:** electron-coupled proton buffer, H_2_ storage and transportation, polyoxometalates, water splitting

## Abstract

We present strategies to tune the redox properties of polyoxometalate clusters to enhance the electron‐coupled proton‐buffer‐mediated water splitting process, in which the evolution of hydrogen and oxygen can occur in different forms and is separated in time and space. By substituting the heteroatom template in the Keggin‐type polyoxometalate cluster, H_6_ZnW_12_O_40_, it is possible to double the number of electrons and protonation in the redox reactions (from two to four). This increase can be achieved with better matching of the energy levels as indicated by the redox potentials, compared to the ones of well‐studied H_3_PW_12_O_40_ and H_4_SiW_12_O_40_. This means that H_6_ZnW_12_O_40_ can act as a high‐performance redox mediator in an electrolytic cell for the on‐demand generation of hydrogen with a high decoupling efficiency of 95.5 % and an electrochemical energy efficiency of 83.3 %. Furthermore, the H_6_ZnW_12_O_40_ cluster also exhibits an excellent cycling behaviour and redox reversibility with almost 100 % H_2_‐mediated capacity retention during 200 cycles and a high coulombic efficiency >92 % each cycle at 30 mA cm^−2^.

## Graphical abstract

**The molecular structure** of keggin‐type tungsten polyoxometalates (POMs) were tuned with different heteroatoms to modify their redox properties. Compared to the two electrons of well‐studied H_3_PW_12_O_40_ and H_4_SiW_12_O_40_, H_6_ZnW_12_O_40_ exhibits double reversible electrons with protonation in the redox reactions and the more desirable redox potentials, which enhance its ECPB performance (see scheme).

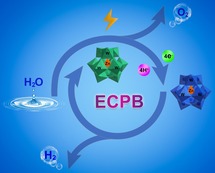

Water splitting is a promising process that could provide a route to a clean and inexhaustible renewable energy, but significant issues remain for establishing scalable systems and also coping with intermittent power.^1^ In this regard proton exchange membrane electrolysis (PEME) is a prime candidate to achieve this transformation but gas crossover through the membrane is both a safety concern and affects the purity of H_2_, as well as degrading the membrane.^2^, ^3^ Thus, it is highly important to develop new strategies to coordinate the intermittent renewable power sources that meet the requirements of H_2_ large‐scale production, transportation and storage simultaneously with safe and economic approaches.

Previously, we introduced the electron‐coupled‐proton‐buffer (ECPB) to separate the process of water splitting in time and space.^4^, ^5^, ^6^, ^7^ In this way, the hydrogen evolution reaction (HER) is no longer coupled with the rate‐limiting step of the oxygen evolution reaction (OER). Electric energy can be stored in a soluble H_2_‐mediated redox couple, which can be chemically or electrochemically converted into gaseous H_2_ on‐demand. Such a system is ideal as a flexible and scalable option to mitigate the intermittent output of renewably generated energy. It will also prevent the crossover issue of O_2_/H_2_ gas through the membrane. Similarly, Xia et al. decoupled the hydrogen and oxygen production system in a membrane‐free electrolyser based on the reversible solid‐state Ni(OH)_2_ and polytriphenylamine redox mediator.^8^, ^9^ Integrated battery‐electrolysers have also been constructed to decouple hydrogen production driven by the electrochemical energy storage system, such as an all‐vanadium dual circuit redox flow battery or a nickel‐iron battery.^10^, ^11^ Furthermore, such hydrogen‐mediated redox species also show potential applications in high‐purity H_2_ storage and generation on demand. Hydrogen could be spontaneously evolved from the reduced‐ECPB on a conventional HER‐catalyst if its redox potential is more negative than the HER onset potential of the catalyst.^12^, ^13^ By exploiting the overpotentials related to hydrogen evolution on carbon, an ECPB can be reduced past the point of the normal hydrogen electrode (NHE) without any H_2_ evolution. Once the reduced ECPB encounters the HER catalyst, it can then be oxidized with concomitant high‐purity H_2_ release—that is, without any additional energy input.

Soluble redox mediators with the ability to buffer protons are the cornerstone of the promising applications described above, but the electron‐storage capacity of the mediators considered hitherto are limited to only 1–2 electrons per molecule. Thus, there is a great need to develop new redox mediators that can store as many electrons per molecule as possible. Polyoxometalates (POMs) show tremendous promise in this regard, due to their ability to perform reversible multielectron reactions with high structural stability in aqueous media.^14]^ Despite many successful demonstrations of polyoxometalates in solid‐state energy storage systems^15^, ^16^, ^17^, ^18^, ^19^, ^20^, ^21^ and redox flow batteries,^22^, ^23^ it is still difficult to develop strategies to modify the POMs’ structure to tune the number of reversible electrons in aqueous states, and thus to increase the volumetric capacities for the applications in ECPB water splitting and hydrogen‐mediated redox couples for aqueous energy storage. Moreover, the energetic inputs required for ECPB‐based water splitting depends on the redox species’ electrochemical potential. For example, silicotungstic acid, can undergo a 2‐electron redox reaction, but only one of the two electrons can be decoupled to release H_2_ spontaneously by exposure over Pt/C catalysts, leading to a low H_2_ decoupling efficiency around 67 % and an electrochemical energy efficiency of 79.3 %.^7^ Thus, it is important to increase the electron storage capacity and to modify the redox potential to improve energy efficiency in the overall water splitting.

Herein, we successfully illustrate the strategies to tune redox properties of Keggin‐type tungsten POMs with different central heteroatoms. The number of reversible electrons with protonation could be doubled by changing the heteroatom from P^5+^or Si^4+^ to Zn^2+^. The desired redox potentials of H_6_ZnW_12_O_40_ enhance its ECPB performance for on‐demand storage and generation of H_2_ with a high decoupling efficiency of 95.5 % and an electrochemical energy efficiency of 83.3 %.

The molecular structure in this work is based on the typical Keggin‐type heteropolyoxotungstates {XW_12_O_40_}. As shown in the CV results in Figure 1 a, {XW_12_O_40_} exhibits distinguished differences of redox chemistry by changing their central heteroatoms (X=P^5+^, Si^4+^, and Zn^2+^) within the voltage range of HER onset potentials between glassy carbon electrode and Pt electrode. This allows {XW_12_O_40_} to be reduced firstly on carbon without any competing H_2_ evolution and then evolve H_2_ spontaneously through simple exposure to Pt later. H_3_PW_12_O_40_ undergoes two redox steps at the potentials +0.222 and −0.052 V vs. NHE. While the redox potentials of H_4_SiW_12_O_40_ shift negatively to +0.019 and −0.212 V vs. NHE compared to those of H_3_PW_12_O_40_. These two separated redox peaks in both H_3_PW_12_O_40_ and H_4_SiW_12_O_40_ have been confirmed to be two one‐electron redox processes.^24^ Intriguingly, due to the prominent effect of ionic charge on the pristine anion clusters, the midpoint potential of the first redox wave shift negatively when the central heteroatom changes from P^5+^ or Si^4+^ to Zn^2+^. H_6_ZnW_12_O_40_ (prepared by following the modified literature method,^25^ see the Supporting Information, Section SI‐2 and Figures S1 and S2) exhibits the redox potentials of −0.078 and −0.198 V vs. NHE under the same conditions. This results are consistent with previous studies of the energy levels.^26^, ^27^, ^28^, ^29^, ^30^ Moreover, the number of electrons stored by H_6_ZnW_12_O_40_ at each redox potential was increased to 2, respectively.

**Figure 1:**
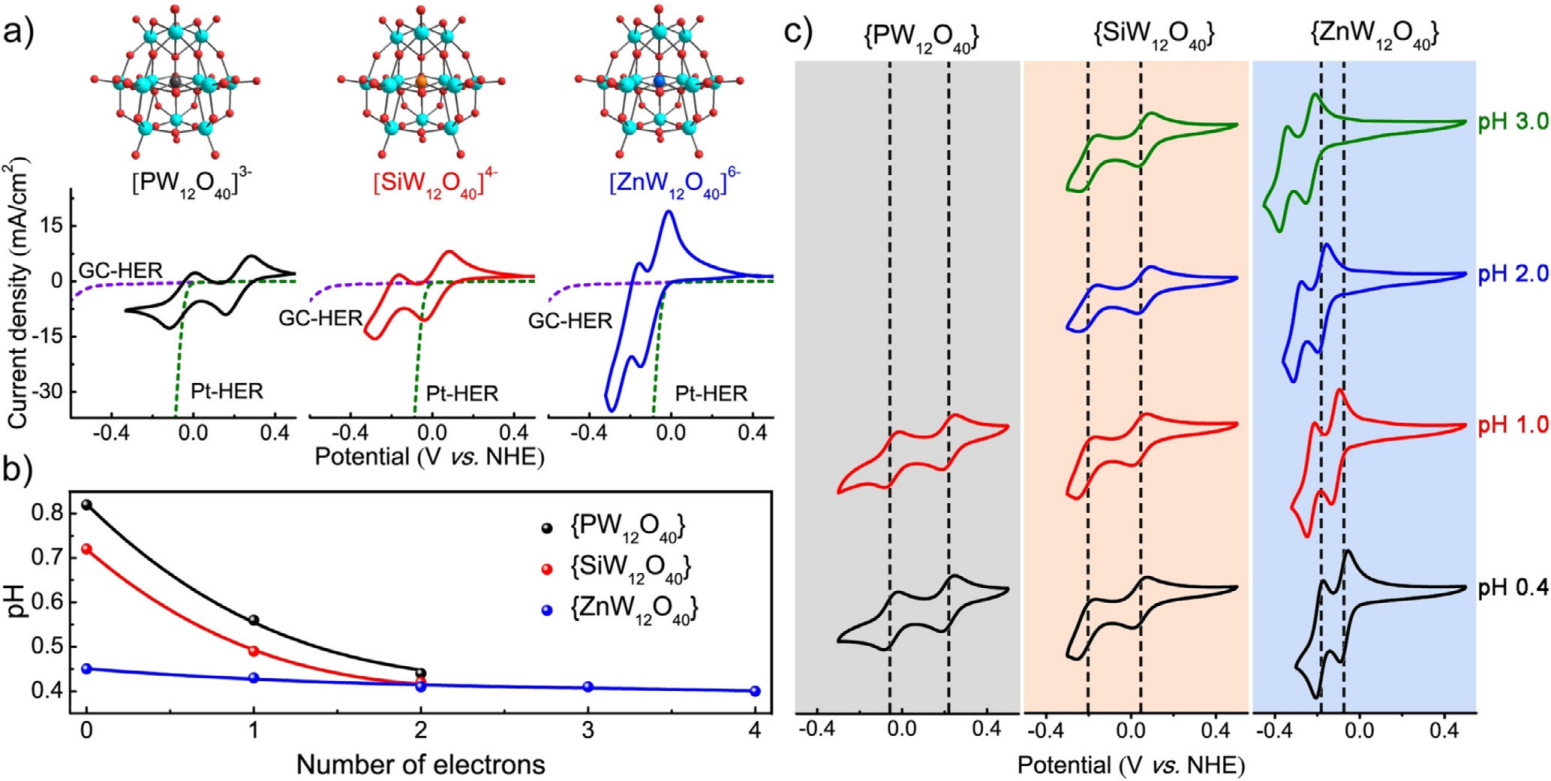
a) Crystal structure of {XW_12_O_40_} (phosphorous, grey; silicon, orange; zinc, blue; tungsten, cerulean; oxygen, red) and the corresponding cyclic voltammograms (CVs) of 100 mm H_n_XW_12_O_40_ (X=P^5+^, Si^4+^, and Zn^2+^) solution on glassy carbon electrode at a scan rate of 50 mV s^−1^. b) pH changing during the reduction process of 100 mm H_n_XW_12_O_40_ (X=P^5+^, Si^4+^, and Zn^2+^) solution. c) CVs of a 1 mm H_*n*_XW_12_O_40_ (X=P^5+^, Si^4+^, and Zn^2+^) under different pH buffer solutions at a scan rate of 50 mV s^−1.^ For more details, see the Supporting Information, Sections SI‐3, SI‐4, and SI‐5.

Changing the heteroatom type inside the {XW_12_O_40_} also leads to a significant difference in ability of the cluster to buffer under reducing conditions. Bulk electrolysis in an airtight H‐cell was performed to electrochemically reduce {XW_12_O_40_} with different equivalents of electrons per cluster (see the details in the Supporting Information, Section SI‐5). As shown in Figure 1 b, a continuous decrease of pH could be observed when 100 mm H_3_PW_12_O_40_ and H_4_SiW_12_O_40_ solution are reduced to two electrons per cluster, while 100 mm H_6_ZnW_12_O_40_ solution has a negligible pH change up to four electron reductions per cluster. This can be ascribed to the enhanced protonation ability of {ZnW_12_O_40_} that accompany reduction in acid solution, keeping the overall ionic charge of the reduced Keggin {XW_12_O_40_} cluster constant at −6.^31^, ^32^, ^33^ This is also confirmed by the CV investigation under different pH values. As shown in Figure 1 c, the redox potential of H_6_ZnW_12_O_40_ has a dependence on the pH of the solution, which means that protons are involved in the electrochemical redox reaction of H_6_ZnW_12_O_40_ according to the Nernst equation. However, the redox potential of H_3_PW_12_O_40_ and H_4_SiW_12_O_40_ has no dependence on pH. The CVs of H_3_PW_12_O_40_ at pH 2.0 and 3.0 cannot be obtained because of its instability in the solution with pH>1.5.^32^

Four‐electron redox reactions of H_6_ZnW_12_O_40_ were further proved by passing charge equivalents to different numbers of electrons per cluster at a current density of 5 mA cm^−2^ and measuring the coulombic efficiency between the reduction and reoxidation process. As shown in Figure 2, the coulombic efficiency remains above 94 % even up to a reduction level of four electrons per cluster molecule. But more than four electrons, reduction of H_6_ZnW_12_O_40_ produces a brown solution, which indicates that some irreversible reactions have occurred (Figure S5, Supporting Information). XPS of the W in the {ZnW_12_O_40_} cluster under the various reduced states are displayed in Figure S6 (see more details of sample preparation in the Supportin Information, Section SI‐7). The peaks at 34.1 and 36.28 eV are assigned to W^V^ 4f_7/2_ and W^Ⅴ^ 4f_5/2_, whereas the peaks in 35.39 and 37.57 eV belong to W^Ⅵ^ 4f_7/2_ and W^Ⅵ^ 4f_5/2_.^34^ The content of W^Ⅴ^ increases obviously with the number of reduction electrons, while the peaks of Zn 2p_3/2_ and Zn 2p_1/2_ at 1021.82 and 1044.88 eV, respectively remain unchanged (Supporting Information, Figure S7), indicating that the negative charges are mainly localized around tungsten sites. Thus, taken all together, H_6_ZnW_12_O_40_ could exhibit a four‐electron redox reaction with accompanying protons and the detailed electrochemical reactions that can occur are presented in the Supporting Information (Section SI‐6). Moreover, the modulated redox potentials make it suitable as an ECPB for high‐performance water splitting.

**Figure 2:**
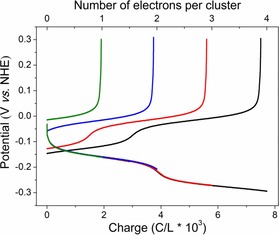
Reduction and reoxidation of a 20 mm H_6_ZnW_12_O_40_ solution with a constant current density of 5 mA cm^−2^ with different equivalents of electrons per cluster.

Regarding the hypothesis of application for on‐demand H_2_ storage and generation, the spontaneous H_2_ evolution experiments were carried out to test the hydrogen‐mediated capacity of H_6_ZnW_12_O_40_. The schematic of this ECPB‐mediated water‐splitting system was illustrated in Figure 3 a. At the anode, H_2_O is split into O_2_, protons, and electrons, whereas the mediator is reversibly reduced and protonated at the cathode in preference to direct production of H_2_. In other words, the electrons were stored in the H_6_ZnW_12_O_40_ by means of electron‐coupled protons rather than direct H_2_ evolution at the cathode. The reduced ECPB is then transferred to a separate chamber for H_2_ evolution over a suitable catalyst. According to their CVs in Figure 1 a, H_6_ZnW_12_O_40_ will be reduced by four electrons and then couple with four protons at the constant potential of −0.36 V vs. NHE. Silicotungstic acid was used as the control experiment. The theoretical H_2_ volume stored in the 2 e^−^‐reduced H_4_SiW_12_O_40_ or 4 e^−^‐reduced H_6_ZnW_12_O_40_ solution could be calculated to 44.8 and 89.6 mL by the charge passed in the first‐step electroreduction process (the calculated equation is given in the Supporting Information, Section SI‐9). As shown in Figure 3 b, about 30 mL H_2_, 67 % of the theoretical H_2_ volume, can be collected by physically mixing the 2 e^−^‐reduced H_4_SiW_12_O_40_ solution with 5 mg 20 % Pt/C, which is consistent with previous reported result.^7^ The incomplete release of hydrogen stored is caused by the first redox potential of H_4_SiW_12_O_40_ being more positive than the HER onset potential of Pt/C catalyst (Figure 1 a). Nevertheless, 95.5 % of the theoretical H_2_ volume stored could be released in the H_6_ZnW_12_O_40_‐mediated system (see movie S1) and the H_2_ initial decoupling rate could reach a value of 121 mmol h^−1^ mg^−1^ Pt, which is much higher than that of the 2 e^−^‐reduced H_4_SiW_12_O_40_. The filtered H_6_ZnW_12_O_40_ solution after hydrogen release shows the same UV/Vis spectrum as the initial solution (Supporting Information, Figure S9), which indicates the structural stability of the H_6_ZnW_12_O_40_ mediator. Moreover, the H_6_ZnW_12_O_40_‐mediated system has a higher electrochemical energy efficiency of 83.3 % than the H_4_SiW_12_O_40_‐mediated system due to its modulated redox potentials and redox electron number (for the detailed calculation process see the Supporting Information, Section SI‐8).

**Figure 3:**
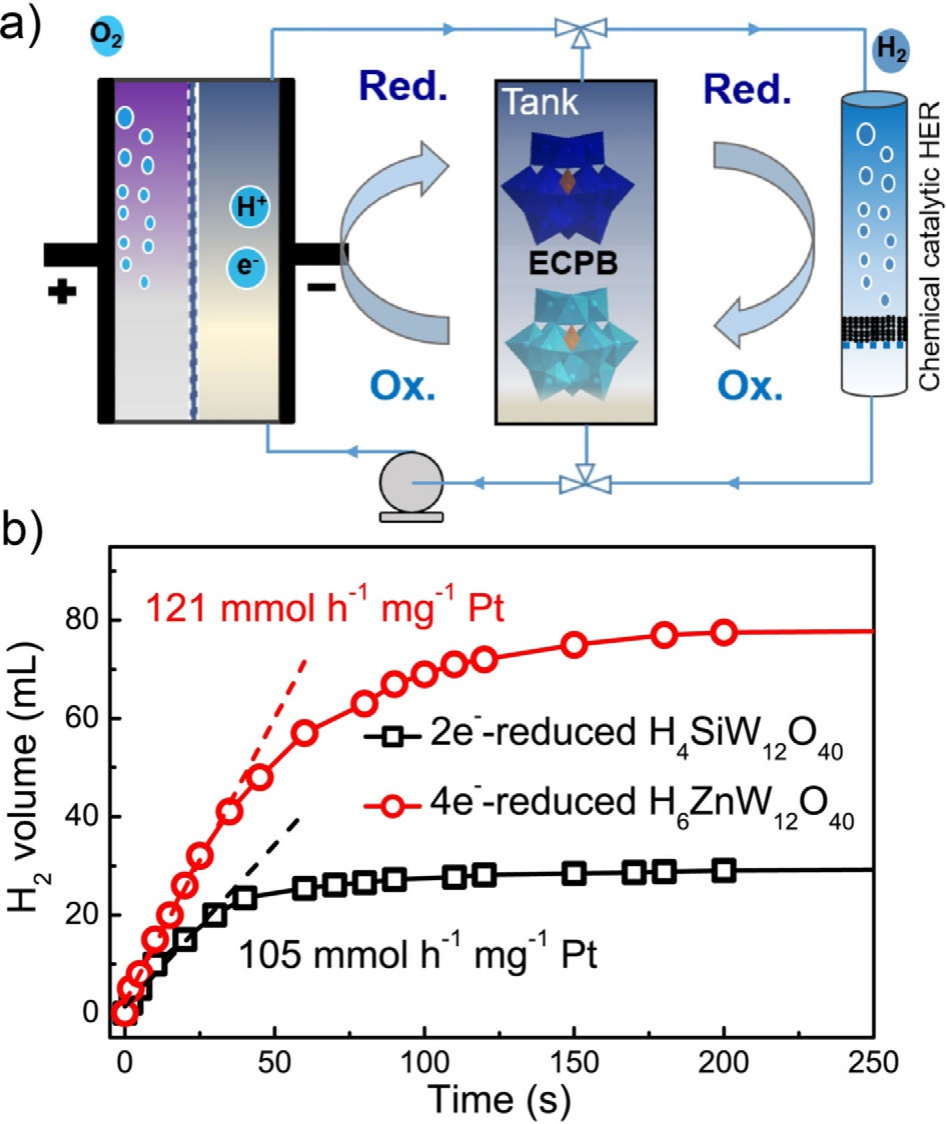
a) Schematic of the ECPB‐mediated water‐splitting system and the spontaneous hydrogen evolution system. b) Spontaneous H_2_ evolution from a 20 mL sample of 100 mm 4 e^−^‐reduced H_6_ZnW_12_O_40_ or 2 e^−^‐reduced H_4_SiW_12_O_40_ over 5 mg 20 % Pt/C.

Another potential application of this ECPB in the production of H_2_ and O_2_ at separated time and space were evaluated by the flow cells system (Figure 4 a). In this system, water is oxidized to produce O_2_ on the anode in cell‐a with an iridium oxide catalyst. The concomitant electrons and protons would be applied to reduce and protonate the H_6_ZnW_12_O_40_ solution on a carbon cathode (12.96 cm^2^ of geometric area) in cell‐a. Once a full 4‐electron reduction per H_6_ZnW_12_O_40_ cluster has been reached by passing corresponding amount of charge, the 4 e^−^‐reduced H_6_ZnW_12_O_40_ solution would be then pumped to a carbon anode in cell‐b to be reoxidized electrochemically and H_2_ is produced on the cathode in cell‐b with a Pt/C catalyst at the same time. The charge passed in the reoxidation process is denoted as practical ECPB storage capacity and the coulombic efficiency could be gauged by comparing it with the charge initially used to reduce H_6_ZnW_12_O_40_ solution. As a result, H_6_ZnW_12_O_40_ exhibits excellent ECPB cycling behaviors and redox reversibility. Under a constant electrolysis current density of 30 mA cm^−2^, it delivers an almost 100 % H_2_‐mediated capacity retention during 200 cycles with a high coulombic efficiency >92 % each cycle (Figure 4 b).

**Figure 4:**
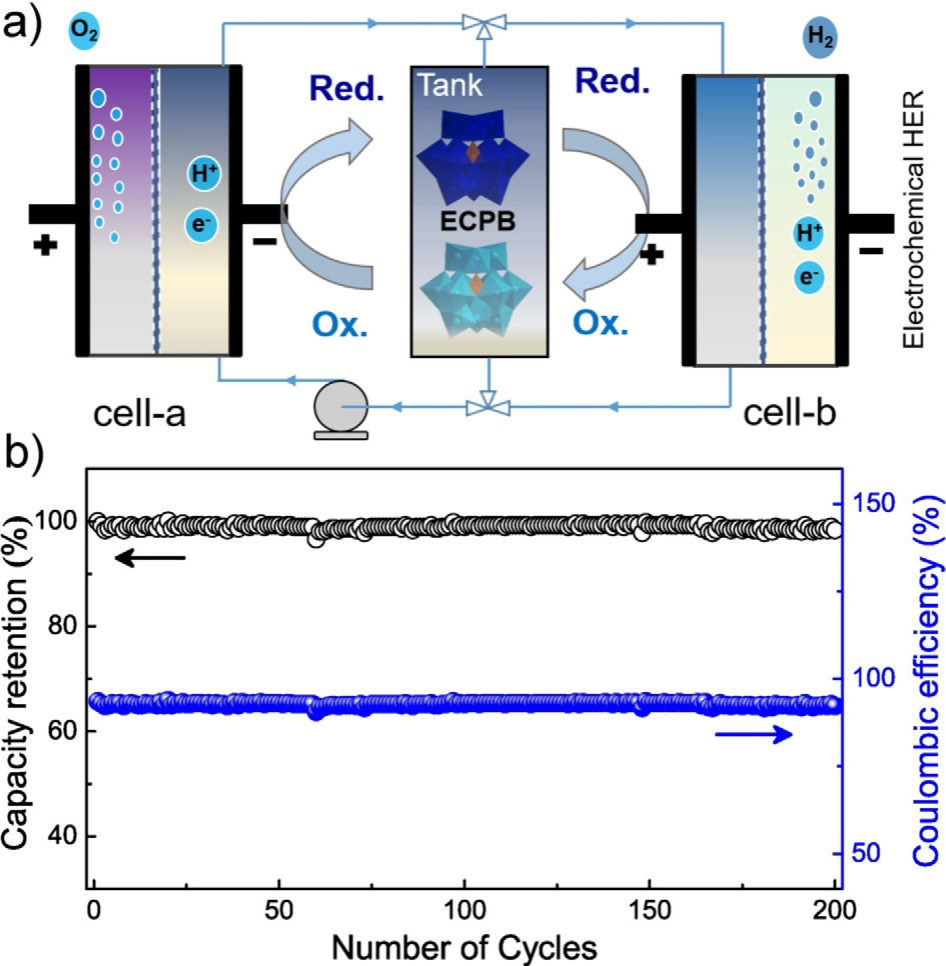
a) Schematic of the flow cells system for long‐term separated time and space water electrolysis. b) Long‐term electrochemical reduction and re‐oxidation cycling of a 20 mm H_6_ZnW_12_O_40_ solution at a current density of 30 mA cm^−2^ under Ar atmosphere.

In summary, this work shows the strategies to tune the properties of H_2_‐mediated redox couples by the adjustment of Keggin‐type tungsten polyoxometalates’ molecular structure with different heteroatoms. When the central heteroatom changes from P^5+^or Si^4+^ to Zn^2+^, H_6_ZnW_12_O_40_ exhibits an increased number of reversible electrons with enhanced protonation ability and more favorable redox potentials. As an ECPB for H_2_ storage and generation, it also delivers a high decoupling efficiency of 95.5 % and an electrochemical energy efficiency of 83.3 % for on‐demand catalytic H_2_ evolution and an excellent long‐term time and space separated, water electrolysis. This work illustrates how to optimize practical application issues by using fundamental approaches, and we believe this will lead to new flexible and safe H_2_ production, storage and transportation systems to mitigate the challenges inherent in present renewable energy systems.

## Conflict of interest

The authors declare no conflict of interest.

## Supporting information

As a service to our authors and readers, this journal provides supporting information supplied by the authors. Such materials are peer reviewed and may be re‐organized for online delivery, but are not copy‐edited or typeset. Technical support issues arising from supporting information (other than missing files) should be addressed to the authors.

Supplementary

Supplementary
