# Supplementary material for: Tuning Redox Active Polyoxometalates for Efficient Electron‐Coupled Proton‐Buffer‐Mediated Water Splitting
Source: Chemistry. 2019 Aug 8;25(49):11432–6. doi: 10.1002/chem.201903142 (PMC6851869; doi:10.1002/chem.201903142)
Supplement: Supplementary file 2 — Supplementary [file CHEM-25-11432-s002.pdf]

CHEMISTRY
A European Journal

Supporting Information

Tuning Redox Active Polyoxometalates for Efficient Electron-Coupled Proton-Buffer-Mediated Water Splitting

Jie Lei,[a] Jun-Jie Yang,[a] Ting Liu,[a] Ru-Ming Yuan,[a] Ding-Rong Deng,[b] Ming-Sen Zheng,[a] Jia-Jia Chen,*[a] Leroy Cronin,*[a, c] and Quan-Feng Dong*[a]

chem_201903142_sm_miscellaneous_information.mp4
